# Thoracic ultrasound for pleural effusion in the intensive care unit: a narrative review from diagnosis to treatment

**DOI:** 10.1186/s13054-017-1897-5

**Published:** 2017-12-28

**Authors:** E. Brogi, L. Gargani, E. Bignami, F. Barbariol, A. Marra, F. Forfori, L. Vetrugno

**Affiliations:** 1 Department of Anaesthesia and Intensive Care, University of Pisa, Via Paradisa 2, 56124 Pisa, Italy; 2 Institute of Clinical Physiology, National Research Council, Pisa, Italy; 3 Department of Anesthesia and Intensive Care, IRCCS San Raffaele Scientific Institute, Milan, Italy; 4 Department of Medicine, University of Udine, Udine, Italy; 5 Department of Neurosciences, Reproductive and Odontostomatological Sciences, University of Naples “Federico II”, Naples, Italy

**Keywords:** Pleural effusion, Lung, Ultrasonography, Catheters, Critical care

## Abstract

Pleural effusion (PLEFF), mostly caused by volume overload, congestive heart failure, and pleuropulmonary infection, is a common condition in critical care patients. Thoracic ultrasound (TUS) helps clinicians not only to visualize pleural effusion, but also to distinguish between the different types. Furthermore, TUS is essential during thoracentesis and chest tube drainage as it increases safety and decreases life-threatening complications. It is crucial not only during needle or tube drainage insertion, but also to monitor the volume of the drained PLEFF. Moreover, TUS can help diagnose co-existing lung diseases, often with a higher specificity and sensitivity than chest radiography and without the need for X-ray exposure. We review data regarding the diagnosis and management of pleural effusion, paying particular attention to the impact of ultrasound. Technical data concerning thoracentesis and chest tube drainage are also provided.

**Electronic supplementary material:**

The online version of this article (doi:10.1186/s13054-017-1897-5) contains supplementary material, which is available to authorized users.

## Take home message

Lung ultrasound is gaining popularity in critical care settings due to its high diagnostic accuracy, its ability to be used at the bedside, and treatment aids. For pleural effusion, lung ultrasound could be essential from diagnosis through clinical management to the final treatment.

## Background

Several medical conditions are responsible for pleural effusion (PLEFF), with volume overload, congestive heart failure, and pleuropulmonary infection representing the most common causes in the intensive care unit (ICU) [1]. Fluid accumulation can take place due to an imbalance of hydrostatic and oncotic pressure across the lung capillaries, increased pleural membrane capillary permeability, and lymphatic obstruction [2]. Furthermore, the factors known to promote lung edema formation in the clinical setting (fluid loading, myocardial depression, hypoalbuminemia) usually coexist, and may exceed the normally high absorptive capacity of the lungs and parietal pleural lymphatics, resulting in exacerbation of PLEFF.

PLEFF occurs frequently in ICU patients with an incidence that varies according to the diagnostic technique used (from 8% following physical examination to more than 60% after routine imaging) [3, 4]. It is also associated with a high crude mortality rate [5]. Indeed, PLEFF can worsen gas exchange, hemodynamic stability, and respiratory dynamics. Drainage can improve oxygenation, respiratory mechanics, and compliance by enhancing the ventilation:perfusion ratio and by re-expanding areas of collapsed parenchymal lung [6, 7]. Although percutaneous pleural drain insertion is frequently carried out in the ICU, inaccurate insertion of the tube can have tragic consequences [8, 9]. Complications with the insertion of chest drains have been reported in up to 20–30% of cases; some of these complications can be potentially fatal (i.e., perforation of the lung, heart, liver, esophagus, spleen, and inferior vena cava) [10].

Prompt diagnosis of the presence and nature of PLEFF is vital in order to evaluate the best therapeutic choice (diuretics, invasive procedures). The use of thoracic ultrasound (TUS), a safe and non-invasive bedside procedure with good accuracy, can help clinicians to visualize the effusion and also to distinguish between different types (exudative, transudative empyema, hemothorax) [11]. Moreover, TUS is important during thoracentesis and the insertion of chest drains as it helps increase safety and decrease life-threatening complications (e.g., organ laceration and infection) [12]. It is fundamental not only during needle or drainage insertion, but also when monitoring the volume of PLEFF drained, and deciding when to remove the drainage tube. Furthermore, TUS can help diagnose co-existing lung diseases, often with higher specificity and sensitivity than chest radiography and without X-ray exposure [13]. Consequently, TUS has become an essential skill for many specialists, especially intensivists [14, 15].

The aim of this review is to summarize current knowledge regarding pleural effusion and chest drainage in ICU patients, focusing on the impact of ultrasound on diagnosis, volume assessment, and drainage techniques in pleural effusion. We have only included trials published after 2000. Technical data concerning thoracentesis and chest tube drainage insertion are also provided.

## Diagnosis of pleural effusion

### Diagnostic imaging

Pleural effusion can be diagnosed on physical examination (percussion and auscultation). The absence of breathing sounds during auscultation, flatness on percussion, and reduced tactile fremitus in patients able to speak are all signs of the presence of PLEFF. However, physical examination can be really challenging in critically ill patients due to the presence of several factors that can alter the intrathoracic transmission of sounds (i.e., mechanical ventilation, bedside position, obese patients, subcutaneous emphysema, uncooperative patients, presence of surgical drains). Consequently, physical examination showed lower sensitivity and specificity for the diagnosis of pleural effusion in comparison to imaging techniques in several trials [13, 16].

**Fig. 1:**
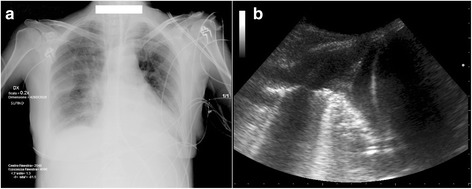
**a** Supine chest X-ray showing a modest right pleural obstruction. **b** The corresponding ultrasound image, showing a pleural effusion of about 2 cm



In order to confirm a suspected diagnosis of PLEFF, physicians can use the following imaging techniques: plain chest radiography (CXR), chest computed tomography (CT), and TUS. CT is considered the gold standard, but it is costly and not always easy to perform on ICU patients [17]. At the bedside, CXR has long been the reference examination for lung imaging. Costophrenic angle blunting, cardiophrenic angle blunting, and lung opacification can indicate PLEFF. However, technical limitations (e.g., supine position, posteroanterior views) and coexisting parenchymal lung disorders might contribute to poor quality X-ray imaging and the underdiagnosis of PLEFF. Effusions as small as 50 mL can be visible in upright lateral CXR images, but conventional posteroanterior images require a volume of at least 200 mL, and volumes of approximately 500 mL obliterate the hemidiaphragm [18]. Lateral decubitus radiography can identify a smaller amount of fluid compared to posteroanterior radiography as costophrenic angles are deeper posteriorly, but erect or lateral decubitus would not be feasible in ICU patients. Ultrasound appears to measure effusion size more accurately than lateral decubitus radiography [19] (Fig. 1). Kocijancic et al. [19] conducted a prospective observational study comparing ultrasound and lateral decubitus radiography to detect PLEFF. They observed that TUS was able to identify pleural effusions smaller than 15 mm. Consequently, several studies focused on the implementation of TUS in the ICU, thus reducing the number of CXR examinations, not only to reduce X-ray exposure, but also to obtain more accurate information [13, 20–22]. Furthermore, a growing body of literature has recognized the superiority of TUS for the diagnosis of PLEFF. Kataoka et al. compared thoracic ultrasound with the physical examination and upright posteroanterior chest radiography for the diagnosis of PLEFF in patients with chronic heart failure [16]. They reported a diagnostic accuracy of 91% for thoracic ultrasound, compared with 56% for the physical examination, and 33% for X-ray examination. In 2004, Lichtenstein et al. [13] carried out a prospective study on 32 patients with acute respiratory distress syndrome (ARDS), as well as ten volunteers, to compare the accuracy of physical examination, TUS, and chest radiography with that of thoracic CT. Lung ultrasound had a sensitivity of 92%, a specificity of 93%, and a diagnostic accuracy of 93%. Bedside chest radiography and physical examination showed lower percentages of sensitivity, specificity, and diagnostic accuracy for pleural effusion [13]. In 2008, Rocco et al. [23] published a trial comparing bedside radiography and ultrasound for PLEFF diagnosis in trauma patients. They showed TUS to be more accurate than radiography for the detection of PLEFF. In 2011, Xirouchaki et al. [24] compared the diagnostic performance of TUS and bedside CXR in ICU patients. For PLEFF diagnosis, TUS showed a sensitivity of 100%, a specificity of 100%, and a diagnostic accuracy of 100%, whereas for CXR these were 65, 81, and 69%, respectively. Consequently, the International Consensus Conference on Lung Ultrasound stated, “for the detection of effusion, lung ultrasound is more accurate than supine radiography and is as accurate as CT” [25]. Notably, TUS can be sometimes useful when defining the nature of PLEFF [26, 27] and in ruling out coexisting lung pathologies (e.g., pneumothorax, atelectasis, alveolar consolidation, interstitial syndrome). Examples of ultrasonography visualization of pleural effusion are available in Additional file 1: video 1, Additional file 2: video 2, Additional file 3: video 3, and Additional file 4: video 4.

**Additional file 1:** Right pleural effusion. A 3.5 MHz probe was chosen and positioned in a longitudinal view at the sixth intercostal space, along the posterior axillary line, with the patient in a semi-recumbent position. It is possible to observe (from *left* to *right*) a small portion of the liver, the echogenic curvilinear diaphragm, and a significant anechoic pleural effusion with associated pulmonary collapse. (MPG 810 kb)




**Additional file 2:** Infra-hepatic view of the right pleural effusion. The probe was positioned between the last ribs on the right side of the patient, under the liver border, oriented toward the patient's nipple, using a transverse scan with the patient in a semi-recumbent position. At the *top* of the image a portion of the liver parenchyma can be seen. It is also possible to visualize the diaphragm (an echoic line that moves with the respiration), the collapsed lung (hyperechoic), and the pleural effusion (anechoic). (MPG 1529 kb)




**Additional file 3:** Pleural effusion and collapsed lung: transverse view. (MPG 1314 kb)




**Additional file 4:** Transverse view of a right pleural effusion at the lung base, along the posterior axillary line. In this intercostal window of the right lower chest, we can see a large pleural effusion with an associated vast lung consolidation. The white hyperechoic spots represent the trapped air inside the collapsed pulmonary parenchyma (air bronchogram). (MPG 905 kb)


### Assessment of fluid volume

**Fig. 2:**
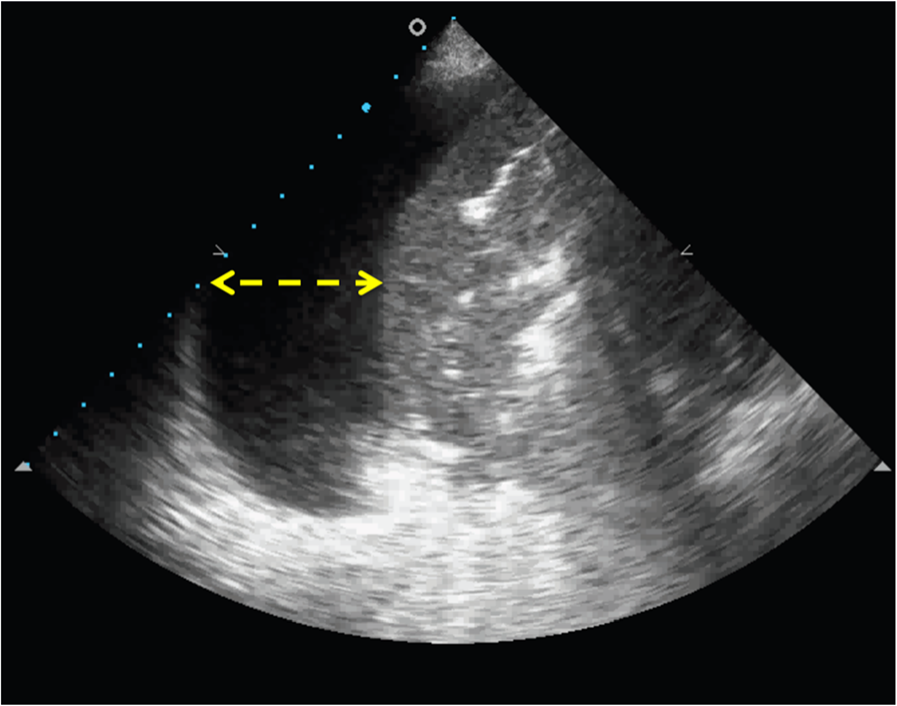
Ultrasound technique to measure the pleural effusion (in centimeters), from chest wall to pulmonary parenchyma (*dashed line*)

Fluid volume, together with alteration in gas exchange, is maybe the most important factor when deciding whether or not to drain a PLEFF. In the case of small effusion volume, the benefits of this procedure should be considered against the risks of complications (pneumothorax or bleeding) [28]. Many ultrasound methods have been proposed to estimate effusion volume (Tables 1 and 2; Fig. 2).

**Table 1:** Assessment of fluid volume

Authors	Patients	Position	Derived formula	Results	Mean prediction error^a^/mean bias^b^	Limits of agreements^c^/standard error^d^
Vignon et al. [29]	Mechanically ventilated and not ventilated patients	Supine	NA	Pleural effusion > 800 mL predicted when the distance is: > 45 mm (right; sensitivity 94%, specificity 76%); > 50 mm (left; sensitivity 100%, specificity 67%) Positive linear correlation between distance and volume: *r* = 088; r^2^ = 0.72; *p* < 0.0001	28 ± 146 mL^b^	−286 mL and +343mL^c^
Roch et al. [30]	Mechanicall ventilated patients	Supine with arm abducted	NA	A distance between lung and posterior chest wall at the lung base > 5 cm predicted a drained volume > 500 mL (sensitivity 83%, specificity 90%, positive predictive value 91%, negative predictive value 82%)	NA	NA
Balik et al. [31]	Mechanically ventilated patients	Supine with mild trunk elevation at 15°	V(ml) = 20 × Sep (mm)	Positive linear correlation between distance (Sep) and volume: *r* = 0.72; r^2^ = 0.52; *p* < 0.001	158.4 ± 160.6^a^	NA
Usta et al. [32]	Spontaneous breathing patients after cardiac surgery	Sitting	V (ml) = 16 × D (mm)	Positive linear correlation between D and V: r = 0.89, r^2^ = 0.79; *p* < 0.001	−21.1 ± 97.78^a^	97.42^d^
Remérand et al. [33]	Critically ill patients	Supine	V (ml) = L_US_ × A_US_	Positive linear correlation between ultrasound V and drained V (r = 0.84, *p* < 0.001) and with CT V (r = 0.90, *p* < 0.001).	−33^b^	−292 to + 227 mL^c^

*A*_*US*_ cross-sectional area, *CT* computed tomography, *D* distance, *L*_*US*_ paravertebral length, *NA* not applicable, *PLEFF* pleural effusion, *Sep* maximal distance between parietal and visceral pleura, *V* pleural volume

^a^Mean prediction error

^b^Mean bias

^c^Limits of agreements

^d^Standard error

**Table 2:** Position of the probe and indications on how to measure pleural effusion by ultrasound, according to different studies

Authors	Probe position	How to measure (end-expiration)
Vignon et al. [29]	Along the dorsolateral part of the chest wall, as posteriorly as possible between the mattress and the patient’s back without lifting the hemithorax, in all IC from the base to the apex	Choose the maximal perpendicular interpleural distance from the leading edge of the dependent surface of the lung to the trailing edge of posterior chest wall, at the apex and at the base
Roch et al. [30]	Along the posterior axillary line between the ninth and eleventh ribs to identify the liver on the right side, the spleen on the left side, and the diaphragm To visualize the effusion, the transducer was advanced cranially and a longitudinal view was chosen	Use the mean of three measurements obtained by distance between: - Lung and diaphragm - Lung and posterior chest wall at base - Lung and posterior chest wall at fifth IC
Balik et al. [31]	Along the posterior axillary line moving the probe cranially, obtaining transverse sections perpendicular to the body axis	Choose the maximal distance between parietal and visceral pleura at lung base (minimum requirement: distance ≥ 10 mm)
Usta et al. [32]	Along mid-scapular line moving cranially (dorsal scanning)	Choose the maximal distance between mid-height of the diaphragm and visceral pleura (minimum requirement: distance ≥ 30 mm)
Remérand et al. [33]	Along each paravertebral intercostal space, slipping the probe between the patient’s back and mattress	The lower and upper intercostal spaces where PLEFF is detected should be drawn on the patient’s skin to establish PLEFF paravertebral length (L_US_) At the half point of L_US_ the PLEFF area should be manually delineated

*IC* intercostal space

**Fig. 3:**
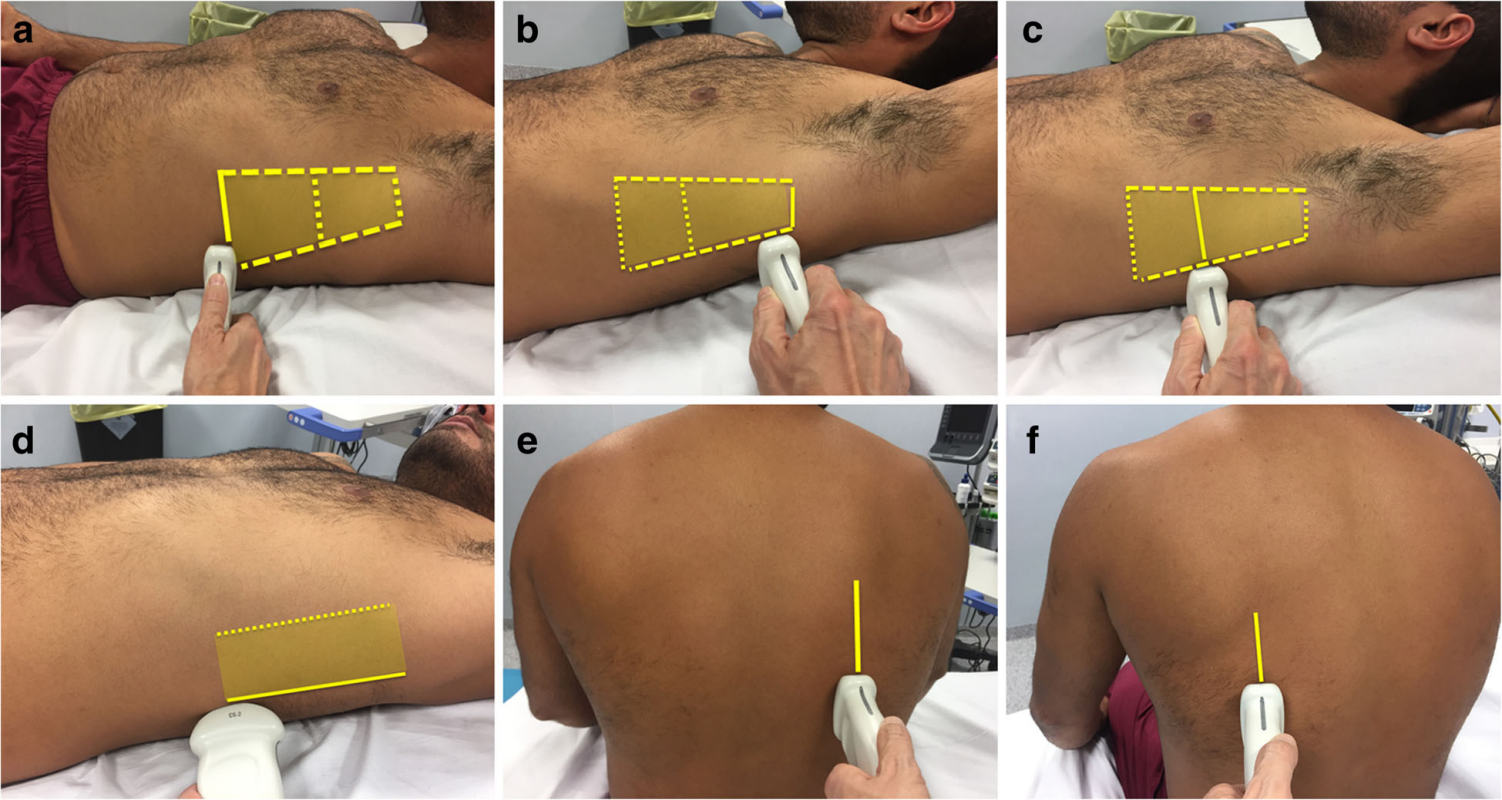
Position of the probe and the marker on the chest wall according to different methods. **a** Supine patient, transverse approach, probe on the posterior axillary line at the lung base/PLEFF lower limit [29–31, 33]. **b** Supine patient, transverse approach, probe on the posterior axillary line at the lung apex/PLEFF upper limit [29, 33]. **c** Supine patient, transverse approach, probe on the posterior axillary line at the middle point of PLEFF height [33]. **d** Supine patient, longitudinal approach, probe on the posterior axillary line to identify the liver on the right side, the spleen on the left side, and the diaphragm [30]. **e** Sitting patient, longitudinal approach, probe on the mid-scapular line on the right lung base [32]. **f** Sitting patient, longitudinal approach, probe on the mid-scapular line on the left lung base [32]

Vignon et al. [29] measured the interpleural distance (the distance between visceral and parietal pleura, or the distance between the lung and posterior chest wall) at the apex and at the lung base, and compared the maximal distance with the drained volume. They found a strong correlation between the interpleural distance and the drained volume. They also found that an interpleural distance of > 45 mm at the right thoracic base or > 50 mm at the left thoracic base predicted PLEFF of > 800 ml. However, the volume prediction appeared to be more accurate for PLEFF of < 1400 mL. Roch et al. [30] performed a study involving 44 patients undergoing mechanical ventilation to assess the accuracy of chest ultrasound in predicting pleural effusions of > 500 ml. They observed that the interpleural distance measured by ultrasound at the lung base or at the fifth intercostal space (PLDbase or PLD5), correlated with the drained volume. Furthermore, a PLDbase of > 5 cm predicted a volume of > 500 mL. Balik et al. [31] measured the maximal interpleural distance (Sep) at end-expiration in 81 mechanically ventilated patients at the lung base (Fig. 2). They found a good correlation between the amount of pleural volume and Sep and suggested that pleural volume could be quantified using the formula: V (ml) = 20 × Sep (mm). According to this study, ultrasound evaluation helps to quantify pleural fluid and to decide whether or not to perform thoracentesis. Usta et al. [32] measured the maximal distance between the mid-height of the diaphragm and the visceral pleura (D) in the sitting position in spontaneously breathing patients after cardiac surgery. They also found a strong correlation between D and the drained volume, and the following formula was derived: V (ml) = 16 × D (mm). Remérand et al. [33] conducted a one-year prospective trial of critically ill patients in order to propose a new technique for pleural effusion volume assessment. The authors identified the lower and upper intercostal spaces where pleural effusion was visible in supine patients; the distance between these two points was drawn on the patient’s skin to establish pleural effusion paravertebral length (L_US_). At the half point of L_US_ the pleural effusion cross-sectional area (A_US_) was manually delineated. Pleural effusion volume was obtained by multiplying L_US_ by A_US._ They observed a strong correlation between ultrasound measurement and both drained volume and CT analysis [33]. The authors conclude that this approach was accurate also when measuring small and moderate PLEFF, and that the measurements of PLEFF depth at the lung base [29–31] can be used only as a rapid screening test to detect a large PLEFF. Probe positioning of these studies with some clues for measurement are summarized in Table 2 and shown in Fig. 3.

There is no established best method among the aforementioned studies. The method proposed by Remerand et al. has the advantage of providing a multiplane approach; on the other hand, the one proposed by Balik et al. seems highly feasible and more reproducible. If the method proposed by Vignon et al. is used, the different cut-off values between the left and right hemithorax should be taken into account (Table 1).

**Fig. 4:**
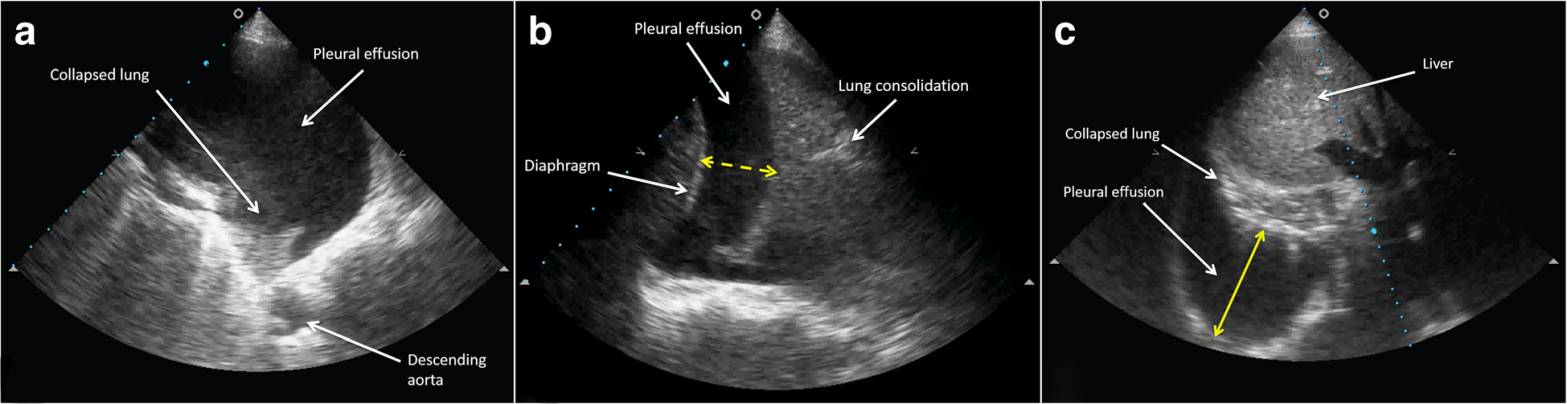
**a** Left pleural effusion. **b** Right pleural effusion. **c** Infra-hepatic view of a right pleural effusion. The main recognizable anatomical structures are marked

Reliable estimation of the effusion volume remains, however, challenging for various reasons. Firstly, ultrasound measurement is influenced by the size of the thoracic cavity. In a tall person with a large thoracic cavity, the amount of fluid is distributed over a larger area than in someone with a smaller thoracic cavity. This can lead to under- or over-estimation of PLEFF. Secondly, a patient’s position (upright, supine, lateral decubitus) can influence fluid distribution. Furthermore, the position of the diaphragm (abdominal hypertension, phrenic nerve paralysis, diaphragmatic hernia) can influence the measurement of PLEFF. Thirdly, in the presence of a very large PLEFF, measurement can be influenced by fluid displacement due to lung collapse (Fig. 4). Moreover, in a very large PLEFF it is not possible to visualize the entire section. Fourthly, the presence of consolidation can influence the shape of fluid accumulation. Fifthly, results can also be influenced by the use of either transverse or longitudinal scans, operator expertise, and inter- and intra-observer variability in the measurement of interpleural distance with ultrasound, estimated to be 6.7–12.8% and 4.8–11.1%, respectively [29]. Transverse scanning tends to overestimate PLEFF measurement, leading to the need for a strict standard ultrasound protocol.

### Technical aspects

#### Ultrasound scanning to define the nature of pleural effusion

**Fig. 5:**
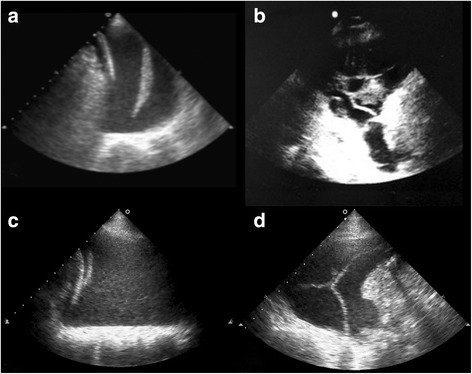
Different types of pleural effusion on ultrasound scan: **a** exudate, **b** empyema, **c** haemothorax, **d** complex septation pleural effusion



What is emerging nowadays is the possible role of ultrasound scanning in the diagnosis of PLEFF. Analysis of PLEFF echogenicity and changes in pleural thickness and lung parenchyma provide important information for the diagnosis of PLEFF (Table 3; Fig. 5). In a prospective study on the possible use of ultrasound for the etiological diagnosis of PLEFF, Yang et al. [11] found that internal echogenicity could be anechoic, complex non-septated, and complex septated (Additional file 5: video 5). The echogenicity of PLEFF can be homogeneous or non-homogeneous (homogenously echogenic spaces). Effusion is defined as anechoic when it is echo-free, as complex non-septated when echogenic material is inside the effusion, and as complex septated when floating fibrin strands or septa are found inside the effusions. The authors concluded that transudates are anechoic, pleural effusions with septation or internal echogenicity are exudates, and that the association of pleural thickness and lung changes also suggests exudates [11]. An anechoic effusion could be either an exudate or a transudate. However, an anechoic bilateral PLEFF would suggest a transudate. Furthermore, homogenously echogenic effusions are typical of hemothorax or empyema (Additional file 6: video 6). In 2004, Sajadieh et al. [26] compared ultrasound findings with laboratory analysis. Ultrasound evaluation of PLEFF consisted of septation, echogenicity, and thickening of the pleura (>3 mm). The authors found that the presence of septation, pleural thickening, and internal echogenicity indicated exudates. An anechoic effusion is more likely to be a transudate. However, exudates too can be visualized as an anechoic area. In the presence of parenchymal lesions, PLEFF is more likely to be an exudate. Moreover, irregular pleural thicknesses are likely to denote malignancy, whereas homogenous echogenic PLEFF characterizes hemothorax or empyema. Other associated ultrasound findings can help with the diagnosis of PLEFF. For example, the presence of pulmonary consolidation (hypoechoic lesion and air bronchograms) is indicative of infection [34]. In 2009, Qureshi et al. [27] evaluated the sensitivity and specificity of thoracic ultrasound in detecting malignant disease in patients with PLEFF. They found that ultrasound was able to discriminate between malignant and benign effusions (overall sensitivity 79%, specificity 100%). Malignancy is characterized by the thickness of the parietal or visceral pleura, the presence of visceral pleural nodules, and diaphragmatic abnormalities (thickness, presence of nodules, layer-resolved). Furthermore, ultrasound scanning can visualize the presence of liver metastases.

**Table 3:** Ultrasound scan to define the nature of pleural effusion

	Exudate	Transudate	Hemothorax	Empyema	Malignant effusion
Internal echogenicity: Anechoic Complex non-septated Complex septated	Septation, non -septated or anaechoic	Anechoic	Hyperechogenic	Often septated	Complex septation, non-septated
Echogenicity: Homogeneity or not	Homogeneity or not	Not	Homogeneity	Homogeneity	Homogeneity
Pleural thickness	Thickened	Normal	Thickness	Thickness	Irregular pleural thickness
Other findings	Based on the etiologies	General bilaterally, echocardiography findings	Pneumothorax, atelectasis, consolidation	Pulmonary consolidation and air bronchograms	Liver metastasis; presence of pleural or diaphragmatic nodules

**Additional file 5:** Transverse view at the lung base, along the posterior axillary line, with complex septatations floating in a massive right pleural effusion. (MPG 1445 kb)




**Additional file 6:** Longitudinal view at the lung base, along the posterior axillary line, with the patient in a semi-recumbent position. Grainy echogenic fluid occupying the left pleural space, due to a massive hemothorax. You can note a small portion of lung consolidation (hyperechoic) near the echogenic curvilinear diaphragm, moving according to the respiratory acts, juxtaposed to the spleen. (MPG 1225 kb)


To summarize, the sonographic characteristics of effusion can help differentiate the nature of PLEFF on the basis of internal echogenicity, homogeneity, and pleural thickness.

#### Probes and puncture site

As suggested by Lichtenstein [35], any kind of ultrasound machine and probe (i.e., phased array, convex, microconvex, and linear) can be used to scan the chest, though a microconvex probe has several advantages for TUS. This kind of transducer is a small ergonomic probe with good spatial resolution and range; using intermediate frequency values, it can visualize both the pleural line and pleural space. Even more, its dimension allows the operator to explore the PLAPS-point (defined as the intersection of a horizontal line at the level of the lower BLUE-point and a vertical line at the posterior axillary line). The PLAPS-point, in fact, is where all free fluids collect in a supine patient; consequently, a scan of this area provides more sensitive detection of PLEFF, even smaller ones. However, this probe is not always available. With regard to the other ultrasound probes (i.e., cardiac, abdominal, and vascular), they have by both advantages and limitations. PLEFF is better visualized using low frequency phased array probes. The abdominal probe (i.e., convex) is ideal for pleural-alveolar characterization, pleural effusion evaluation, and assessment of artifacts; however, it is usually bulky and it may be difficult to explore the PLAPS-point with using it. Cardiac probes (i.e., phased array) are successfully used to detect PLEFF, but sometimes do not clearly show lung sliding [36]. Vascular probes (i.e., linear), with their higher frequency, are ideal for pleural line and subpleural space evaluation; however, the assessment of lung artifacts and pleural effusions is not ideal with this probe [37]. In the absence of a microconvex probe, Lichtenstein suggested using the abdominal probe, recognizing that it may become limited in areas of difficult access or in the evaluation of superficial resolution (i.e., lung sliding assessment) [36]. This suggestion is totally in line with the international recommendations for point-of-care lung ultrasound. In fact, Volpicelli et al. [25] recommended using a microconvex probe as the first choice when evaluating PLEFF. However, when the aforementioned probe is unavailable, it is suggested to select a phased array or a convex transducer. Ideally, the probe should be small enough to be placed in the intercostal spaces with good spatial resolution and range. Generally, a convex probe incorporates these advantages, allows good visualization of the lung, and is widely available in several ultrasound machines [38].

Ultrasound guidance helps to locate the insertion site and prevent organ perforation. Diacon et al. [39] compared ultrasound and physical examination to identify the pleural puncture site for thoracentesis. Ultrasound increased the number of accurate sites detected (presence/absence and thickness of the pleural effusion) by 26% compared to chest percussion, and prevented potential complications in 15% of clinically determined puncture sites [39]. Three characteristics were associated with puncture site inaccuracy: small effusions (*p* < 0.001), radiological evidence of fluid loculation (*p* ≤ 0.01), and sharp costodiaphragmatic angle on CXR (*p* < 0.001). Several other studies confirm the superiority of ultrasound to detect the best puncture site and reduce complications (e.g., pneumothorax) [40–43]. Consequently, The British Thoracic Society pleural disease guideline 2010 concluded that, “site selection for all pleural aspiration should be ultrasound guided” [12].

#### Thoracentesis technique

Thoracentesis is a percutaneous procedure with valuable diagnostic and therapeutic applications. As a diagnostic procedure, in the presence of PLEFF of unknown etiology, thoracentesis allows the nature of the PLEFF to be defined (i.e., differential diagnosis between transudate vs. exudate) and can detect possible etiologies (i.e., malignancy, chylous, infection). As a therapeutic procedure, thoracentesis enables removal of a large volume of fluid and consequently to relieve symptoms of dyspnea in the presence of a sizeable PLEFF [44].

For thoracentesis, patients can be placed, when possible, in an upright sitting position with arms elevated, or in a supine position with an arm behind the head. In this position, effusion gravitates down to the lower part of the chest, leading to an increased safety margin (depth of pleural effusion) [45, 46]. Ultrasound assistance in pleural procedures is performed using either “site marking” or “direct needle guidance” [40]. In the first case, the physician determines the optimal location point and marks it on the skin, and then carries out the procedure without using the ultrasound probe. It is important to note that patient repositioning can lead to fluid redistribution, so drain insertion has to be performed immediately after site marking. For direct needle guidance, the correct position of the needle is visualized in real-time and is monitored constantly. Direct needle guidance is technically more challenging and did not seem to be a safer procedure in comparison to the site marking technique. In fact, Mayo et al. [47] performed 232 ultrasonography-guided thoracentesis procedures in patients receiving mechanical ventilation without using real time needle guidance and reported a really low rate of complications (1.3%). Consequently, real time needle guidance is not necessarily required if site marking is performed correctly.

Pleural aspiration should be a full aseptic technique using a small-bore needle, a syringe, and a tubing system. A needle is inserted into the intercostal space at the superior margin of the inferior rib so as not to damage the neurovascular bundle. In order to prevent re-expansion pulmonary edema (RPE), no more than 1.5 L should be aspirated. Aspiration should be terminated should the patient develop a cough or complain of chest discomfort [12].

#### Chest-drain insertion technique

Indications for chest drain insertion for PLEFF include the following [48]: malignant pleural effusion; empyema or parapnemonic effusion, chylothorax, treatment with sclerosing agent or pleurodesis, recurrent PLEFF, traumatic hemopneumothorax, esophageal rupture with gastric leak into pleural space, and post-surgery.

Chest-drain insertion should be carried out in the *safe triangle* and performed under image guidance [12]^,^. The triangle of safety is bordered by the lateral edge of the latisimus dorsi, the lateral border of the pectoralis muscle, and a line along the fifth intercostal space at the level of the nipple. Localization of the safe triangle is recommended during an emergency procedure, especially when ultrasound is not available (e.g., out-of-hospital emergency care), although ultrasound technology is nowadays widespread in the hospital setting. Indeed, TUS not only allows more precise localization and quantification of PLEFF, but can also detect loculated fluid or anatomical variations (e.g., cardiomegaly, elevated diaphragm, rib metastasis). Consequently, TUS is essential also in emergency situations in order to avoid serious complications (i.e., pneumothorax, bleeding, organ injury). Furthermore, it was once thought that ultrasound was unable to detect intercostal vessels and was therefore incapable of reducing the incidence of vessel laceration [12]. However, some trials have shown how Doppler ultrasound can be used to visualize intercostal vessels [49–52], making it an essential aid for the prevention of vessel laceration.

As recommended by the British Thoracic Society pleural disease guidelines in 2010, blunt dissection should be used in the presence of trauma or the insertion of large-bore catheters. Drainage insertion via blunt dissection poses significantly fewer risks and is the chest drainage technique-of-choice in a surgical context [12]. The major complications linked to large-bore catheters are pain, lung laceration, and infection [53, 54]. Trocars are no longer recommended as they can lead to complications and damage involving the underlying viscera [55].

#### Small-bore catheter insertion

Pigtail catheter insertion is an effective and safe procedure for the drainage of different kinds of PLEFF (massive transudative PLEFF, parapneumonic effusion, malignant PLEFF, postoperative PLEFF, and traumatic hemothorax) [56–58]. The main complications linked to small-bore catheters are blockage, dislodgement, malposition, and kinking [59].

**Fig. 6:**
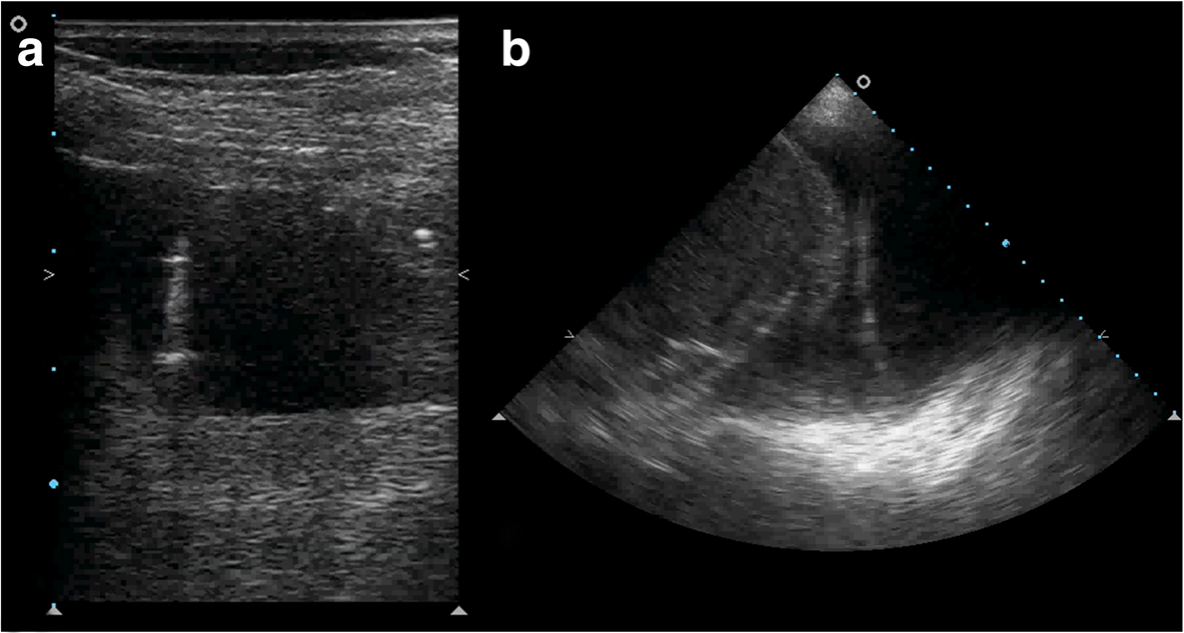
Right pleural effusion seen through a linear (**a**) and convex (**b**) ultrasound probe. The hyperechoic line visible within the pleural hypoechoic effusion is the metallic wire inserted to guide the introduction of the pleural drainage



Small-bore catheter insertion using the Seldinger technique is an easier and less invasive method than traditional thoracotomy tubes [60]. The first part of the Seldinger procedure is identical to thoracentesis. A needle is inserted into the intercostal space and aspiration of fluid with a syringe confirms the correct position using the site marking technique. Unlike in thoracentesis, a guidewire is then inserted through the needle and the needle itself is withdrawn. Ultrasound allows the operator to monitor the insertion and define its final position. Vertical rotation of the probe over the intercostal space allows visualization of the guidewire (leading towards the costophrenic space). At this point, a dilatator is inserted over the guidewire. The dilatator should not be introduced more than 1 cm between the skin and the pleural space; excessive dilatator insertion increases the risk of visceral injury [61]. Some manufacturers include a “collar” around the dilatator to reduce such an occurrence. However, TUS allows the measurement of the chest wall in order to calculate how far the dilatator can be introduced, avoiding complications. Using the linear probe, it is possible to properly measure the distance between the skin and the parietal pleural. Finally, the drain is passed over the guidewire. The appearance of the wire with ultrasonography is shown in Fig. 6 and Additional file 7: video 7. After the guidewire has been removed, the drain is connected to the drainage system. At the end of the procedure it is mandatory to perform a complete bilateral lung ultrasound scan to exclude possible complications (e.g., pneumothorax).

**Additional file 7:** Transverse view at the right lung base, showing a pleural effusion and the moving diaphragm juxtaposed to the liver. The hyperechoic line visible within the pleural hypoechoic effusion is the metallic wire inserted to guide the introduction of the pleural drainage. (MPG 1543 kb)


## Conclusions

Lung ultrasound is a simple non-invasive bedside procedure, with better sensitivity and specificity than chest radiography, for the diagnosis of PLEFF. It is not only crucial for visualizing the effusion, but can also help distinguish between different forms of PLEFF. The use of ultrasound to guide thoracentesis and to insert chest tubes has recently been advocated to increase the safety of this invasive procedure, especially in ventilated ICU patients, or for small, loculated effusions. Furthermore, TUS is also essential to monitor the volume of PLEFF drained, and to decide when to remove the drainage.

## Abbreviations

CT: Computed tomography
CXR: Plain chest radiography
ICU: Intensive care unit
PLD5: Anteroposterior diameter of a non-loculated PLEFF, measured using ultrasound at the fifth intercostal space (or PLD5)
PLDbase: anteroposterior diameter of a non-loculated PLEFF, measured using ultrasound at the lung base
PLEFF: Pleural effusion
RPE: Re-expansion pulmonary edema
Sep: Effusion volume measured the maximal distance between parietal and visceral pleura
TUS: Thoracic ultrasound.
